# Metastatic patterns and survival outcomes in patients with stage IV colon cancer: A population‐based analysis

**DOI:** 10.1002/cam4.2673

**Published:** 2019-11-06

**Authors:** Jiwei Wang, Song Li, Yanna Liu, Chunquan Zhang, Honglang Li, Bin Lai

**Affiliations:** ^1^ Department of Ultrasound The Second Affiliated Hospital of Nanchang University Nanchang China; ^2^ Mudanjiang Medical College Mudanjiang China; ^3^ Department of Gastrointestinal Surgery The Second Affiliated Hospital of Nanchang University Nanchang China

**Keywords:** mucinous adenocarcinoma, prognosis, signet ring cell carcinoma, survival analysis, synchronous metastasis

## Abstract

**Background:**

The goal of this study was to delineate the patterns of distant metastasis from colon adenocarcinoma (CAC) and evaluate the survival differences by metastatic patterns.

**Methods:**

Using the Surveillance, Epidemiology, and End Results (SEER) database, we extracted patients diagnosed with stage IV CAC between 2010 and 2016. Kaplan‐Meier survival curves were plotted with log‐rank tests to compare overall survival (OS) of patients with different metastatic patterns. Univariate and multivariate Cox proportional hazards regression models were used to evaluate the effects of different metastatic patterns on survival outcomes in terms of OS and disease‐specific survival (DSS).

**Results:**

A total of 26 170 patients were analyzed. The 3‐ and 5‐year OS were 20.7% and 10.5%, respectively, for patients with stage IV CAC. The most common distant metastatic site was the liver, followed by the lung, bone, and brain, but the frequency differed greatly by histology subtypes. The site of metastasis was a significant prognostic factor for OS and DSS in patients with stage IV CAC, independent of the number of metastatic sites and other clinical and demographic prognostic factors. Using liver‐only metastasis as reference, lung‐only metastasis was associated with better OS (hazard ratio [HR] = 0.82, 95% confidence interval [CI], 0.71‐0.94) and DSS (HR = 0.75, 95% CI, 0.64‐0.88). Older age, black race, unmarried status, grade III/IV tumors, advanced tumor‐node‐metastasis (TNM) stage, proximal colon, elevated preoperative carcinoembryonic antigen (CEA), no surgery of the primary site, and no chemotherapy were independent predictors of poor OS.

**Conclusions:**

The site of distant metastasis and number of metastasis site were independent prognostic factors for survival of patients with stage IV CAC. This study highlights the need for diverse treatment strategies for patients with different metastatic patterns.

## Graphical abstract

Site of distant metastasis is an independent prognostic factor for survival of patients with metastatic colon cancer. Surgery of the primary tumor and chemotherapy offer significant survival benefits for patients with metastatic disease.

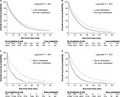

## 1 INTRODUCTION

Colorectal cancer (CRC) is one of the most common cancer worldwide, with over 1.8 million new cases estimated to be diagnosed in 2018.^1^ The United States is among the countries with high incidence of CRC. An estimated 145 600 new CRC cases are expected to occur in 2019, accounting for 8.3% of all new cancers in the United States.^2^ Survival improvement of CRC has been observed in the past decades, largely attributing to the expending efforts in screening and early detection and recent advances in systemic and local treatment modalities. Despite these, CRC remains the third leading cause of cancer‐related mortality in the United States, where an estimated 51 020 deaths from CRC are expected in 2019.^2^ Metastases are the main cause of CRC‐related mortality. Approximately, 22% of CRCs are metastatic at initial diagnosis, and about 70% of patients will eventually develop metastatic relapse.^3^, ^4^, ^5^ Patients with metastatic CRC face poor prognosis in general, with a relative 5‐year survival rate of 14%, compared to 71% and 90% in those with regional and localized CRC in the United States.^6^

Understanding metastatic characteristics with prognostic value is crucial for planning and decision‐making regarding selecting appropriate preventive and therapeutic regimens, but predicting survival outcomes of metastatic CRC remains challenging given the heterogeneity observed in tumor spread and biological features of the primary and metastatic tumors of the colorectum.^7^ The American Joint Committee on Cancer (AJCC) tumor‐node‐metastasis (TNM) classification is widely used for CRC staging and prognosis prediction. The eighth edition of the TNM for CRC, released in 2017, introduced a modification to the M category by including a new M stage involving metastasis to the peritoneum, based on accumulating evidence supporting it as an indicator of poor prognosis.^8^ However, the current M category is still broad, covering a heterogeneous group of metastatic CRCs in terms of survival outcomes and potential effective treatment strategies.

The most frequent anatomic site for metastasis from CRC is the liver, due to its close proximity to the colorectum, with less frequent metastasis to the lung, bone, and nervous system. Considerable differences in site distribution of metastasis have been observed between colon and rectal cancers, and between different histological types.^9^, ^10^ It is well recognized that metastatic spread to more than one distant organ confers worse survival outcomes in patients with CRC; this prognostic indictor has been incorporated into the AJCC TNM classification since the seventh edition published in 2010.^11^ However, whether site of distant metastasis predicts survival outcomes remains unclear with inconsistent evidence.^12^, ^13^, ^14^, ^15^, ^16^, ^17^ Most of these studies focused on a specific metastatic site, mostly the liver, but the prognostic implication of metastatic patterns including other less common sites has not yet been well characterized. In addition, demographic and clinical prognostic factors were rarely taken into consideration when interpreting the differential survival, primarily due to limited sample size and availability of demographic and clinical data.

In this study, we sought to delineate the patterns of distant metastasis and determine whether the site of metastasis correlates with survival outcomes among patients with colon cancer. Colon adenocarcinoma (CAC) is the most common type of colon cancer accounting for more than 90% of colon cancers diagnosed in the United States and was thus selected for the present study.^6^ For this study, we evaluated data from 18 population‐based cancer registries in 14 states that participate in the Surveillance, Epidemiology, and End Results program (SEER 18), which accounts for an approximately 28% of the United States population.^18^

## 2 MATERIALS AND METHODS

### 2.1 Data source

We retrieved data from the latest version of the SEER 18 registries database, released in November 2018, with the SEER*Stat software (version 8.3.5).^19^ SEER is generally considered the gold standard for data quality in cancer registry, with near‐complete case ascertainment and microscopic confirmation.^18^ The SEER 18 database encompasses cancers diagnosed since 2000 and provides follow‐up information regarding survival status and death causes to the end of year 2016 in the most recent version. Since data from SEER are publicly available and de‐identified, this study was exempt from local institutional review board review.

### 2.2 Patent selection

This study included adult patients (≥18 years old) with stage IV CAC diagnosed between 2010 and 2016. Patients not diagnosed as the first or only primary colon cancer were excluded. The diagnosis of CAC was identified using the International Classification of Disease for Oncology, Third Edition (ICD‐O‐3) histology codes for adenocarcinoma (8140‐8147, 8210‐8211, 8220‐8221, 8260‐8263), mucinous adenocarcinoma (MAC) (8480‐8481), and signet ring cell carcinoma (SRCC) (8490) with the colon (site code: C18.0 and C18.2‐18.9) listed as the primary site. The diagnosis was microscopically confirmed and cases identified from autopsy or death certification only were excluded.

### 2.3 Covariates

Data regarding demographics (sex, race, ethnicity, age at diagnosis, and marital status), tumor characteristics (primary site, histologic grade, and AJCC stages), sites of metastasis, treatment, and follow‐up for survival (survival months, vital status, and cause of death) were collected from the SEER database. Race in SEER is coded as white, American Indian/Alaskan, and Asian/Pacific Islander. The latter two were grouped together as “other” in subsequent analysis due to small sample size. The primary tumor sites were categorized as proximal colon (C18.0, C18.2‐18.5), distal colon (C18.6‐18.7), and other (C18.8‐18.9). SEER began to routinely collect carcinoembryonic antigen (CEA) laboratory interpretation prior to treatment for CRC since 2004.^20^ CEA in SEER is coded as negative/normal, borderline (undermined if positive or negative), and positive/elevated. The extent and sites of metastasis were determined from the site‐specific metastasis and AJCC M category data. SEER adopted AJCC seventh edition since 2010 and classified metastasis to one site as category M1a and metastases to multiple sites or peritonea metastasis as category M1b. Site‐specific metastasis data were available in the SEER database since 2010 and only metastasis to the liver, lung, bone, and brain at diagnosis were provided.

### 2.4 Outcome measures

Overall survival (OS) was defined as the survival interval from the time of cancer diagnosis to the time of death reported in vital status in the SEER database. Patients surviving past 31 December 2016 were classified as censored. Death occurring within 30 days of diagnosis was recorded as 0 for survival in months in the SEER database, which was determined considering the use of 30 days as a cut‐off for perioperative mortality. For disease‐specific survival (DSS), deaths from causes other than colon cancer were treated as censored observations.

### 2.5 Statistical analysis

Descriptive statistics were presented as percentage or median with interquartile range as appropriate. A Chi‐square test was used to compare difference between groups for categorical variables. Kaplan‐Meier survival curves were used to plot overall survival and a log‐rank test was used to compare survival curves between patients with different metastasis patterns. A life table analysis was performed to calculate survival rates and corresponding 95% confidence intervals (95% CIs). A Cox proportional hazards regression model was used to calculate hazard ratios (HRs) and 95% CIs of covariates associated with OS and DSS, respectively. Covariates subject to univariate Cox regression analysis included: age at diagnosis (≤median and >median), sex (male and female), race (White, Black, and other), ethnicity (Hispanic, non‐Hispanic), marital status (married, single, widowed, and divorced/separated), primary site (proximal, distal, and other), histologic grade (well differentiated, moderately differentiated, and poor differentiated or undifferentiated), histology (nonmucinous adenocarcinoma, MAC, and SRCC), M category (M1a, M1b, M1NOS), liver metastasis (yes and no), lung metastasis (yes and no), bone metastasis (yes and no), brain metastasis (yes and no), metastasis to one site (liver, lung, brain, bone, and other), CEA (positive, negative), surgery of the primary site performed as part of the first course of treatment (yes, no/unknown), and chemotherapy performed as part of the first course of treatment (yes, no/unknown). These covariates were chosen based on knowledge of possible association with colon cancer occurrence and mortality. For multivariate Cox proportional hazards analysis, a stepwise procedure, with *P* < .15 as the criterion for entry and *P* > .05 as the criterion for removal, was used to select covariates for final multivariate models. A two‐sided *P* < .05 was considered significant. Kaplan‐Meier and log‐rank analyses were performed using GraphPad Prism 7.0 (GraphPad Software). All other tests were performed using SAS 13.2 (SAS Institute Inc).

## 3 RESULTS

### 3.1 Demographic and clinical characteristics

Overall, 26 170 adult patients diagnosed with primary stage IV CAC were reported in the SEER 18 database from 2010 to 2016, with a median (95% CI) OS of 13 months.^13^, ^14^ MAC and SRCC were identified in 2131 (8.14%) and 564 (2.16%) of these patients, with a median (95% CI) OS of 13^12^, ^13^, ^14^ and 8 months,^7^, ^8^, ^9^ respectively. Using life table analysis, OS rates at 3 years and at 5 years were 20.7% (95% CI, 20.1%‐21.2%) and 10.5% (95% CI, 10.0%‐11.0%), respectively, for patients with stage IV CAC. The 3‐year OS rate of patients with metastatic MAC was 17.7% (95% CI, 15.8%‐19.5%), significantly higher than that of patients with metastatic SRCC (7.0%, 95% CI, 4.4%‐9.6%) (*P* < .001).

Table 1 presents the descriptive statistics of demographic and clinical features of these patients. The median age at diagnosis was 64 years and 7.58% of patients with stage IV CAC were younger than 45 years. Slightly more than half of patients were female for CAC and the MAC histology subtype, whereas for SRCC, there were more male than female cases; the difference was significant (*P* < .001). The primary tumor of both MAC and SRCC occurred more frequently in the proximal colon (62.04% and 64.89%, respectively), as compared to CAC overall (53.36%, *P* < .001). The percentage of patients negative for preoperative CEA was 22.70% in patients with SRCC, higher than that in other histological types (*P* < .001). For the choice of treatment, 39.1% of patients received both surgery and chemotherapy as the first course of treatment, followed by chemotherapy only in 24.9% of patients and surgery only in 18.3% of patients. Treatment was more aggressive for patients with metastatic MAC, of whom 46.4% received both surgery and chemotherapy, showing significant difference from SRCC and nonmucinous adenocarcinomas (*P* < .001).

**Table 1:** Demographic and clinical characteristics of patients with stage IV colon adenocarcinoma

Characteristics	Overall (%) (n = 26 170)	MAC (%) (n = 2131)	SRCC (%) (n = 564)
Age (years)^a^	64 (55‐74)	65 (54‐75)	64 (51‐73)
Sex			
Male	13 625 (52.06)	1014 (47.58)	316 (56.03)
Female	12 545 (47.94)	1117 (52.42)	248 (43.97)
Race			
White	19 300 (73.75)	1646 (77.24)	457 (81.03)
Black	4454 (17.02)	330 (15.49)	66 (11.70)
Other	2349 (8.98)	150 (7.04)	41 (7.27)
Unknown	67 (0.26)	5 (0.23)	0
Ethnicity			
Hispanic	3138 (11.99)	264 (12.39)	70 (12.41)
Non‐Hispanic	23 032 (88.01)	1867 (87.61)	494 (87.59)
Marital status			
Married	12 949 (49.48)	1018 (47.77)	293 (51.95)
Single	5252 (20.07)	411 (19.29)	115 (20.39)
Divorced/separated	3044 (11.63)	269 (12.62)	52 (9.22)
Widowed	3544 (13.54)	331 (15.53)	78 (13.83)
Unknown	1381 (5.28)	102 (4.79)	26 (4.61)
Primary site			
Proximal	13 965 (53.36)	1322 (62.04)	366 (64.89)
Distal	9491 (36.27)	528 (24.78)	135 (23.94)
Other	2714 (10.37)	281 (13.19)	63 (11.17)
Tumor grade			
Well differentiated (I)	996 (3.81)	119 (5.58)	4 (0.71)
Moderately differentiated (II)	12 973 (49.57)	938 (44.02)	18 (3.19)
Poorly or undifferentiated (III/IV)	5874 (22.44)	525 (24.64)	398 (70.57)
Unknown	6327 (24.18)	549 (25.76)	144 (25.53)
T stage			
T0‐2	2583 (9.87)	116 (5.44)	37 (6.56)
T3‐4	16 118 (61.59)	1599 (75.04)	382 (67.73)
TX	7469 (28.54)	416 (19.52)	145 (2.57)
N stage			
N0	7897 (30.18)	529 (24.82)	143 (25.35)
N1	7946 (30.36)	642 (30.13)	97 (17.20)
N2	7077 (27.04)	761 (35.71)	244 (43.26)
NX	3250 (12.42)	199 (9.34)	80 (14.18)
Preoperative CEA			
Positive	15 284 (58.40)	1165 (54.67)	277 (49.11)
Borderline	52 (0.20)	3 (0.14)	2 (0.35)
Negative	3137 (11.99)	298 (13.98)	128 (22.70)
Unknown	7697 (29.41)	668 (31.35)	157 (27.84)
Surgery			
Yes	15 009 (57.35)	1515 (71.09)	320 (56.74)
No/unknown	11 161 (42.65)	616 (28.91)	244 (43.26)
Chemotherapy			
Yes	16 752 (64.01)	1352 (63.44)	357 (63.30)
No/unknown	9418 (35.99)	779 (36.56)	207 (36.70)

Abbreviations: CEA, carcinoembryonic antigen; MAC, mucinous adenocarcinoma; SRCC, signet ring cell carcinoma

a Median (interquartile range)

Table 2 describes the results of univariate analysis of survival outcomes stratified by demographic and clinical features in patients with stage IV CAC. On the univariate analysis, significant variables for OS and DSS included age at diagnosis, race, ethnicity, marital status, primary site of the tumor, tumor grade, histology types, TNM stage, preoperative CEA, surgery, and chemotherapy. These factors were entered into a Cox proportional hazards model for multivariate analysis and those remained significant were considered as confounders in the final models evaluating the impact of metastatic patterns on survival.

**Table 2:** Univariate analysis of demographic and clinical features associated with survival outcomes in patients with stage IV colon adenocarcinoma

Characteristics	OS		DSS	
	HR (95% CI)	*P*	HR (95% CI)	*P*
Age (> 64 vs ≤ 64)				
≤ 64	Ref		Ref	
> 64	1.64 (1.59‐1.69)	<.001	1.58 (1.54‐1.64)	<.001
Sex				
Male	Ref		Ref	
Female	1.01 (0.98‐1.04)	.60	1.02 (0.99‐1.05)	.26
Race				
White	Ref		Ref	
Black	1.08 (1.04‐1.12)	<.001	1.07 (1.03‐1.11)	.002
Other	0.90 (0.86‐0.95)	<.001	0.90 (0.85‐0.95)	<.001
Ethnicity				
Hispanic	Ref		Ref	
Non‐Hispanic	1.06 (1.01‐1.11)	.002	1.10 (1.05‐1.16)	<.001
Marital status				
Married	Ref		Ref	
Single	1.19 (1.14‐1.24)	<.001	1.17 (1.13‐1.22)	< 0.001
Divorced/separated	1.15 (1.10‐1.20)	<.001	1.13 (1.07‐1.19)	<.001
Widowed	1.70 (1.63‐1.78)	<.001	1.66 (1.59‐1.74)	<.001
Primary site				
Proximal	Ref		Ref	
Distal	0.73 (0.71‐0.75)	<.001	0.71 (0.68‐0.73)	<.001
Other	1.68 (1.61‐1.76)	<.001	1.37 (1.30‐1.45)	<.001
Tumor grade				
Well differentiated (I)	Ref		Ref	
Moderately differentiated (II)	0.91 (0.84‐0.98)	.012	0.91 (0.83‐0.99)	.024
Poorly or undifferentiated (III/IV)	1.36 (1.25‐1.47)	<.001	1.35 (1.24‐1.47)	<.001
Histology				
Nonmucinous	Ref		Ref	
MAC	1.05 (1.00‐1.11)	.052	1.06 (1.00‐1.12)	.049
SRCC	1.55 (1.41‐1.70)	<.001	1.45 (1.31‐1.62)	<.001
T stage				
T0‐2	Ref		Ref	
T3‐4	0.72 (0.69‐0.76)	<.001	0.75 (0.71‐0.79)	<.001
N stage				
N0	Ref		Ref	
N1	0.77 (0.74‐0.80)	<.001	0.81 (0.77‐0.84)	<.001
N2	0.84 (0.81‐0.87)	<.001	0.91 (0.87‐0.95)	<.001
M stage				
M1a	Ref		Ref	
M1b	1.57 (1.53‐1.62)	<.001	1.59 (1.54‐1.65)	<.001
Preoperative CEA				
Negative	Ref		Ref	
Positive	1.64 (1.55‐1.72)	<.001	1.58 (1.49‐1.67)	<.001
Surgery				
No/unknown	Ref		Ref	
Yes	0.44 (0.43‐0.45)	<.001	0.45 (0.44‐0.47)	<.001
Chemotherapy				
No/unknown	Ref		Ref	
Yes	0.33 (0.32‐0.34)	<.001	0.35 (0.34‐0.35)	<.001

Abbreviations: CEA, carcinoembryonic antigen; CI, confidence interval; DSS, disease‐specific survival; HR, hazard ratio; MAC, mucinous adenocarcinoma; OS, overall survival; SRCC, signet ring cell carcinoma.

### 3.2 Site of metastasis

Table 3 shows the distribution of site of metastasis by M category and histology type. The liver was the most common site of metastasis among these four sites recorded in the SEER data, but the frequency of liver metastasis varied greatly by histology types. Liver metastasis was detected in 73.63% of patients with CAC overall; the percentage reduced to 51.71% in patients with MAC and 20.57% in patients with SRCC. In patients with liver metastasis, solitary metastasis was reported in 54.03% and 47.28% of patients with CAC and with MAC, respectively, comparably higher than 23.28% in patients with SRCC. The lung was second to the liver as a common site of metastasis, detected in 21.76%, 14.17%, and 6.91% of patients with CAC, MAC, and SRCC, respectively. In patients with SRCC, the bone was slightly more common than the lung as a site of metastasis (7.27% and 6.91%, respectively). Concurrent metastases to the liver and lung were detected in 4262 (76.0%) patients with lung metastasis. Metastases to the brain were rare, detected in less than 2% of patients with metastatic disease.

**Table 3:** Sites of distant metastasis for patients with stage IV colon adenocarcinoma

	Site of metastasis	Total (%)	M category		
			M1a	M1b	M1NOS
Overall	Liver	19 268 (73.63)	10 406 (54.03)	8168 (42.41)	694 (3.56)
	Lung	5695 (21.76)	747 (13.11)	4650 (81.65)	298 (5.23)
	Bone	1301 (4.97)	89 (6.84)	1127 (86.63)	85 (6.53)
	Brain	348 (1.33)	61 (17.53)	257 (73.85)	30 (8.62)
MAC‐only	Liver	1102 (51.71)	521 (47.28)	542 (49.18)	39 (3.53)
	Lung	302 (14.17)	37 (12.25)	248 (82.12)	17 (5.63)
	Bone	80 (3.75)	7 (8.75)	66 (82.50)	7 (8.75)
	Brain	13 (0.61)	2 (15.38)	10 (76.92)	1 (1.18)
SRCC‐only	Liver	116 (20.57)	27 (23.28)	83 (71.55)	6 (5.17)
	Lung	39 (6.91)	4 (10.26)	32 (82.05)	3 (7.69)
	Bone	41 (7.27)	6 (14.63)	26 (63.41)	9 (26.47)
	Brain	9 (1.60)	1 (11.11)	7 (77.78)	1 (11.11)

Abbreviations: MAC, mucinous adenocarcinoma; SRCC, signet ring cell carcinoma.

### 3.3 Site‐specific metastasis and survival outcomes

**Figure 1:**
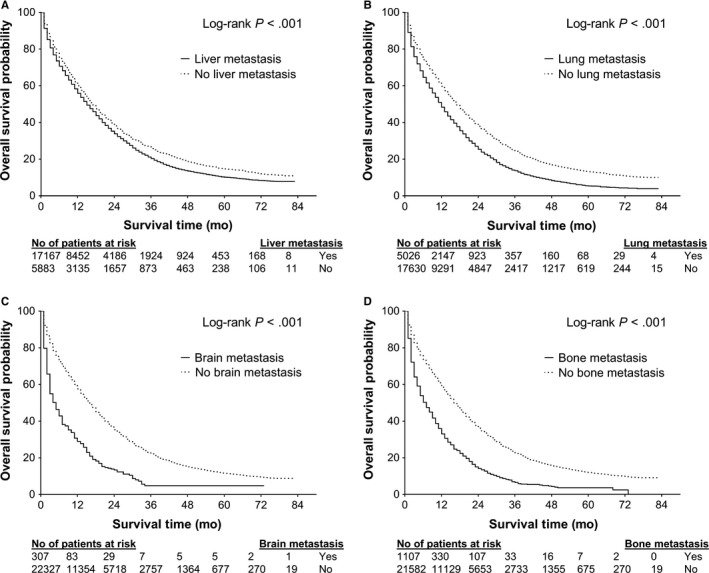
Kaplan‐Meier overall survival curves of patients with stage IV colon adenocarcinoma stratified by the presence of (A) liver metastasis, (B) lung metastasis, (C) brain metastasis, and (D) bone metastasis

To explore the relationship between metastatic patterns and survival, we grouped patients based on the presence of site‐specific metastasis regardless of the number of metastatic sites. Figure 1 displays Kaplan‐Meier curves for OS in patients with and without site‐specific metastasis. Tables 4 and 5 show univariate and multivariate analyses, respectively, of survival outcomes with respect to these metastatic site‐related categories. As illustrated in Figure 1A, the presence of liver metastasis was an adverse prognostic factor for OS (log‐rank *P* < .001). The 3‐year OSs were 19.4% (95% CI, 18.7%‐20.0%) and 24.9% (95% CI, 23.7%‐26.1%) for patients with and without liver metastasis, respectively. On the univariate analysis, the presence of liver metastasis was associated with worse survival in terms of OS and DSS, respectively, in patients with CAC. On the multivariate analysis, the presence of liver metastasis was an independent prognostic factor associated with worse OS (HR = 1.40, 95% CI, 1.32‐1.47) and DSS (HR = 1.46, 95% CI, 1.38‐1.55) in patients with CAC. The liver metastasis‐specific HRs retained significant in patients with MAC.

**Table 4:** Univariate analysis of metastasis patterns associated with survival outcomes in patients with stage IV colon adenocarcinoma

Site of metastasis	Overall				MAC‐only				SRCC‐only			
	OS		DSS		OS		DSS		OS		DSS	
	HR (95% CI)	*P*	HR (95% CI)	*P*	HR (95% CI)	*P*	HR (95% CI)	*P*	HR (95% CI)	*P*	HR (95% CI)	*P*
Liver (Yes vs No)	1.18 (1.14‐1.22)	<.001	1.24 (1.20‐1.29)	<.001	1.26 (1.14‐1.40)	<.001	1.36 (1.22‐1.52)	<.001	1.39 (1.11‐1.74)	.004	1.41 (1.09‐1.82)	.009
Lung (Yes vs No)	1.39 (1.34‐1.43)	<.001	1.40 (1.35‐1.45)	<.001	1.30 (1.14‐1.49)	<.001	1.27 (1.10‐1.48)	.002	2.04 (1.47‐2.85)	<.001	2.05 (1.41‐3.00)	<.001
Bone (Yes vs No)	1.88 (1.77‐2.00)	<.001	1.84 (1.71‐1.97)	<.001	2.24 (1.77‐2.84)	<.001	2.31 (1.78‐2.99)	<.001	1.94 (1.38‐2.74)	<.001	1.92 (1.29‐2.85)	.001
Brain (Yes vs No)	1.95 (1.74‐2.19)	<.001	1.86 (1.64‐2.12)	<.001	1.90 (1.05‐3.44)	<0.034	1.70 (0.85‐3.41)	.134	1.13 (0.56‐2.28)	.107	1.15 (0.51‐2.59)	.745
One site of metastasis												
Liver‐only	Ref		Ref		Ref		Ref		Ref		Ref	
Lung‐only	0.82 (0.74‐0.90)	<.001	0.75 (0.68‐0.84)	<.001	0.60 (0.39‐0.94)	.024	0.50 (0.30‐0.84)	.009	1.88 (0.63‐5.56)	.255	1.69 (0.49‐5.87)	.696
Bone‐only	1.43 (1.10‐1.86)	.007	1.28 (0.95‐1.73)	.111	1.11 (0.49‐2.48)	.803	0.87 (0.32‐2.33)	.869	2.14 (0.79‐5.82)	.134	1.09 (0.25‐4.79)	.912
Brain‐only	1.53 (1.15‐2.04)	.003	1.59 (1.16‐2.16)	.004	2.97 (0.74‐11.95)	.125	3.64 (0.90‐14.66)	.069	‐		‐	
Other unspecified	0.79 (0.74‐0.85)	<.001	0.75 (0.70‐0.81)	<.001	0.71 (0.58‐0.87)	<.001	0.66 (0.53‐0.82)	<.001	1.03 (0.60‐1.76)	.920	0.85 (0.47‐1.56)	.599

Abbreviations: CI, confidence interval; DSS, disease‐specific survival; HR, hazard ratio; MAC, mucinous adenocarcinoma; OS, overall survival; SRCC, signet ring cell carcinoma

**Table 5:** Multivariate analysis of metastasis patterns associated with survival outcomes in patients with stage IV colon adenocarcinoma

Site of metastasis	Overall				MAC‐only			
	OS		DSS		OS		DSS	
	HR (95% CI)	*P*	HR (95% CI)	*P*	HR (95% CI)	*P*	HR (95% CI)	*P*
Liver (Yes vs No)	1.40 (1.32‐1.47)	<.001	1.46 (1.38‐1.55)	<.001	1.39 (1.19‐1.63)	<.001	1.44 (1.22‐1.71)	<.001
Lung (Yes vs No)	1.11 (1.05‐1.17)	<.001	1.11 (1.05‐1.19)	<.001	—		—	
Bone (Yes vs No)	1.30 (1.18‐1.45)	<.001	1.27 (1.13‐1.42)	<.001	1.94 (1.21‐3.10)	.006	1.96 (1.19‐3.22)	.008
Brain (Yes vs No)	1.36 (1.14‐1.63)	<.001	1.31 (1.07‐1.60)	.008	—		—	
One site of metastasis								
Liver‐only	Ref		Ref		Ref		Ref	
Lung‐only	0.82 (0.71‐0.94)	.005	0.75 (0.64‐0.88)	<.001	0.66 (0.3‐1.24)	.197	0.41 (0.19‐0.91)	.027
Bone‐only	1.06 (0.67‐1.66)	.810	0.88 (0.51‐1.53)	.658	0.68 (0.09‐4.96)	.706	1.01 (0.14‐7.27)	.994
Brain‐only	1.49 (1.04‐2.13)	.032	1.49 (1.00‐2.22)	.048	15.99 (2.15‐118.9)	.007	18.53 (2.45‐139.9)	.027
Other unspecified	0.62 (0.56‐0.69)	<.001	0.60 (0.54‐0.67)	<.001	0.60 (0.45‐0.81)	<.001	0.55 (0.40‐0.76)	<.001

Abbreviations: CI, confidence interval; DSS, disease‐specific survival; HR, hazard ratio; MAC, mucinous adenocarcinoma; OS, overall survival.

Metastasis to the lung, bone, and brain also had significant independent prognostic value in patients with stage IV CAC, but only presence of bone metastasis retained as an independent prognostic factor for OS and DSS in patients with MAC (Table 4 and 5). In patients with CAC, the 3‐year OSs were 12.9% (95% CI, 11.9%‐14.0%) for patients with lung metastasis and 23.3% (95% CI, 22.6%‐24.0%) for patients without lung metastasis, 8.7% (95% CI, 7.1%‐10.4%) for patients with bone metastasis and 21.7% (95% CI, 21.1%‐22.4%) for patients without bone metastasis, and 5.7% (95% CI, 2.6%‐8.7%) for patients with brain metastasis and 21.2% (95% CI, 20.5%‐21.8%) for patients without brain metastasis.

### 3.4 Site of solitary metastasis and survival outcomes

**Figure 2:**
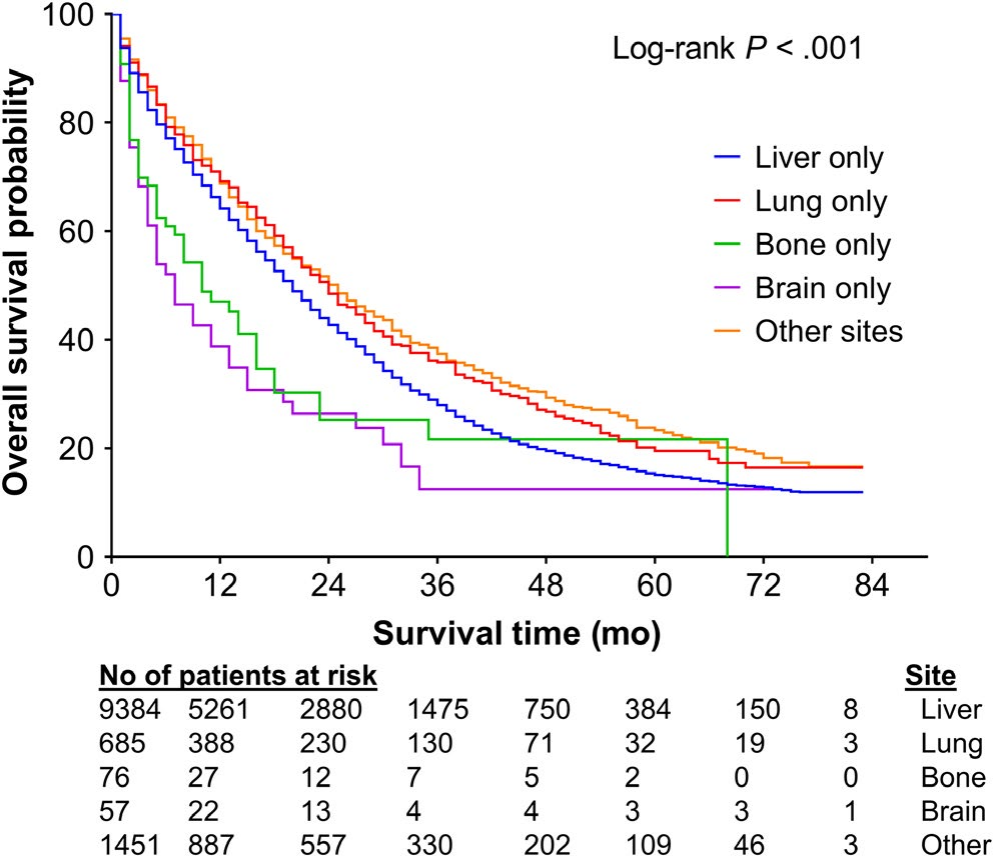
Kaplan‐Meier overall survival curve of patients with M1a colon adenocarcinoma stratified by site of metastasis

A subgroup analysis in patients with M1a disease was performed to evaluate the impact of site of solitary metastasis on survival outcomes. In the subgroup analysis, we grouped patients into five categories based on site of metastasis and used liver‐only metastasis as the reference based on relatively large sample size of this group. Patients with M1a disease but without metastasis to the liver, lung, bone, or brain were categorized into other unspecified sites. As illustrated in the Kaplan‐Meier curves in Figure 2, the survival probability differed significantly among these sites (log‐rank *P* < .001). Using life table analysis, 3‐year OSs for patients with solitary metastasis to the liver, lung, bone, brain, and other unspecified sites were 26.8% (95% CI, 25.8%‐27.8%), 34.0% (95% CI, 29.9%‐38.1%), 21.6% (95% CI, 10.8%‐32.5%), 14.0% (95% CI, 3.6%‐24.4%), and 36.2 (95% CI, 33.5%‐39.0%), respectively. Multivariate Cox proportional hazards analysis revealed that compared with liver‐only metastasis, lung‐only metastasis and solitary metastasis to other unspecified sites were independent prognostic indicators for better survival in terms of both OS (HR = 0.82, 95% CI, 0.71‐0.94; HR = 0.62, 95% CI, 0.56‐0.69, respectively) and DSS (HR = 0.75, 95% CI, 0.64‐0.88; HR = 0.60, 95% CI, 0.54‐0.67, respectively) (Table 5). In contrast, brain‐only metastasis was associated with worse survival compared with liver‐only metastasis. The significantly worse OS in patients with bone‐only metastasis compared with liver‐only metastasis found on univariate analysis disappeared after multivariate adjustment for possible confounding variables. In patients with MAC, brain‐only metastasis had a more pronounced prognostic value on survival.

### 3.5 Prognostic factors for patients with solitary metastasis to the liver and lung

Considering the relatively small sample size of patients with solitary metastasis to other sites, we only identified prognostic factors that were associated with OS for patients with solitary metastasis to the liver and lung, respectively (Table 6). In both groups, married status was significantly associated with better OS. For patients with liver‐only metastasis, distal colon as the primary tumor site was associated with better survival, but such prognostic benefit was not observed in patients with lung‐only metastasis. Metastasis in four or more regional lymph nodes (N2 stage) independently predicted worse survival, while T stage did not act as an independent factor in affecting OS in patients with liver‐only or lung‐only metastasis. Elevated preoperative CEA was associated with worse survival for patients with liver‐only (HR = 1.61, 95% CI, 1.46‐1.78) and lung‐only metastasis (HR = 1.37, 95% CI, 1.00‐1.87). Notably, surgery of the primary site performed as part of the first course of treatment significantly improved OS both in patients with liver‐only metastasis (HR = 0.37, 95% CI, 0.33‐0.40) and lung‐only metastasis (HR = 0.27, 95% CI, 0.19‐0.40). Similarly, chemotherapy performed as the first course of treatment significantly prolonged survival of patients with liver‐only metastasis (HR = 0.30, 95% CI, 0.28‐0.32) and with lung‐only metastasis (HR = 0.30, 95% CI, 0.23‐0.40).

**Table 6:** Multivariate analysis of factors associated with overall survival in patients with solitary metastasis to the liver and lung

Variables	Liver‐only		Lung‐only	
	HR (95% CI)	*P*	HR (95% CI)	*P*
Age (year)				
≤ 64	Ref		—	
> 64	1.35 (1.26‐1.45)	<.001	—	
Race				
White	Ref		—	
Black	1.11 (1.02‐1.22)	.017	—	
Other	0.84 (0.75‐0.95)	.006	—	
Marital status				
Married	Ref		Ref	
Single	1.10 (1.01‐1.21)	.024	1.62 (1.10‐2.38)	.014
Divorced/separated	1.07 (0.97‐1.18)	.196	1.49 (0.98‐2.28)	.064
Widowed	1.22 (1.10‐1.35)	<.001	2.52 (1.73‐3.68)	<.001
Primary site				
Proximal	Ref		—	
Distal	0.75 (0.70‐0.80)	<.001	—	
Other	1.26 (1.05‐1.50)	.012	—	
Tumor grade				
Well differentiated (I)	Ref		Ref	
Moderately differentiated (II)	0.93 (0.80‐1.09)	.373	1.30 (0.69‐2.43)	.416
Poorly or undifferentiated (III/IV)	1.47 (1.25‐1.74)	<.001	2.12 (1.06‐4.27)	.035
N stage				
N0	Ref			
N1	1.21 (1.11‐1.33)	<.001	0.92 (0.65‐1.30)	.635
N2	1.53 (1.39‐1.69)	<0.001	1.56 (1.07‐2.29)	.021
Preoperative CEA				
Negative	Ref		Ref	
Positive	1.61 (1.46‐1.78)	<.001	1.37 (1.00‐1.87)	.049
Surgery				
Yes	Ref		Ref	
No/unknown	0.37 (0.33‐0.40)	<.001	0.27 (0.19‐0.40)	<.001
Chemotherapy				
Yes	Ref		Ref	
No/unknown	0.30 (0.28‐0.32)	<.001	0.30 (0.23‐0.40)	<.001

Abbreviations: CEA, carcinoembryonic antigen; CI, confidence interval; HR, hazard ratio.

## 4 DISCUSSION

Recent progress in understanding metastasis at molecular and genetic level has arouse growing interest in the epidemiology of metastatic CRC. In this study, we evaluated the association between metastatic patterns and survival outcomes of colon cancer using the SEER 18 registries data. Our findings demonstrated significant prognostic value of the site of distant metastasis at diagnosis in patients with stage IV CAC, which was independent of the number of metastatic sites and other clinical and demographic prognostic factors affecting survival. In patients with solitary metastasis, lung‐only metastasis was a favorable prognostic indicator of OS and DSS compared with liver‐only metastasis. In addition, we identified independent prognostic factors for patients with liver‐only metastasis and lung‐only metastasis and uniformly found a strong survival benefit of surgery of the primary site and chemotherapy as the initial treatment of choice.

Accumulated studies compared the survival of CRC with different metastatic sites, but most studies were based on single institution experience with relatively small sample size or focused on a specific site of metastasis.^14^, ^17^, ^21^, ^22^ The analysis reported herein took advantage of the large patient population of the SEER data to comprehensively examine the impact of metastatic sites on survival outcomes with adjustment for possible demographic and clinical variables known to affect survival. Consistent with previous literatures in small patient populations,^23^, ^24^, ^25^ we confirmed that lung‐only metastasis had survival advantage over liver‐only metastasis even after multivariate adjustment. A recent analysis of two Australian cancer registry databases (5967 patients with CRC) revealed that the median OSs of brain‐only and bone‐only metastasis were much lower than that of liver‐only and lung‐only metastasis in patients with CRC.^15^ Our univariate analysis results are largely in line with this report in Austrian patients, but multivariate adjustment eliminated the survival disadvantage of bone‐only metastasis compared with liver‐only metastasis. This seems against the common expectation that bone metastasis has a poor prognosis, but the results need to be explained in more detail. Firstly, the observed survival difference between patients with bone‐only and liver‐only metastasis may be explained by confounders. In our study, multiple demographic and clinical covariates affecting survival were included in the final multivariate Cox model. In an analysis of 1207 patients with CRC, the survival disadvantage of bone‐only metastasis over liver‐only metastasis was statistically significant but substantially diminished after adjustment with age and treatment only, supporting the significance of confounding.^23^ Second, our analysis showed that 86.63% of patients with bone metastasis were in M1b stage, higher than the percentage of patients with metastasis to other distant organs. This is in line with previous case‐series studies indicating that bone‐only metastasis was a rare event for patients with colon cancer.^26^, ^27^ Bone‐only metastasis might reflect a separate entity in terms of tumor spread because metastasis to bone typically occurs through hematogenous dissemination and thus usually accompanies by the metastasis to other organs, predominantly the liver.^28^ However, it should be noted that given the relatively small number of patients with bone‐only metastasis, the multivariate analysis applied in this study may be too conservative or lack enough power to distinguish bone‐only metastasis from liver‐only metastasis in terms of survival outcomes. More studies in larger patient populations are needed to clarify this issue.

MAC and SRCC are uncommon histological subtypes of CAC displaying different clinical profiles and related to worse prognosis than nonmucinous adenocarcinomas.^9^, ^29^, ^30^ In this study, synchronous liver metastasis was detected in 76.89% of patients with nonmucinous adenocarcinoma, but only in 51.71% of patients with MAC and 20.57% of patients with SRCC. In addition, in patients with liver metastasis, solitary metastasis occurred in about half of the patients with nonmucinous adenocarcinoma and with MAC, whereas in only 23.28% of patients with SRCC. These observed differences in metastatic patterns between different histological subtypes of CAC support the seed and soil hypothesis, which suggests that tumors metastasize preferentially to certain organs based on the interaction between tumor cells and microenvironment of their respective target organs.^31^ In accordance with previous studies,^9^, ^30^ survival difference was significant among these adenocarcinoma subtypes, with SRCC having the worst survival, followed by MAC. The clinical profiles of patients with MAC and SRCC included primary tumor to the proximal colon, and advanced T, N, and M stage, which not surprisingly predicted poor survival on both univariate and multivariate analyses. These factors, however, could not explain the survival difference since the survival difference retained significant after adjustment for these factors (results not shown). Notably, multivariate analysis revealed that brain‐only metastasis was a strong prognostic predictor for poor survival of patients with MAC. But this result needs to be interpreted with caution as brain‐only metastasis was reported in only two patients with MAC.

Our study demonstrated a significant survival benefit from surgical resection of the primary tumor and chemotherapy in patients with stage IV colon cancer. The effects remained significant and strong on both univariate and multivariate analyses and in all subgroup analyses by histological types and metastatic patterns. This finding is in line with previous studies that demonstrated primary tumor resection and chemotherapy independently associated with better survival in patients with unresectable synchronous metastases from CRC.^32^, ^33^, ^34^ Our study showed that 17.71% of patients with stage IV colon cancer received neither surgery nor chemotherapy. Therefore, our findings argue for more widespread use of surgical resection of primary tumor and chemotherapy in patients with stage IV disease. But it should be noted that the observed survival benefit related to surgery and chemotherapy may be due to selection bias, that is, patients who present with unresectable lesions or are critically ill may be less likely to receive surgery and chemotherapy as the first line of treatment. In addition, it should be noted that the treatment information in the SEER database is limited by the uncertainty whether patients categorized as “no/unknown” is due to not receiving treatment or due to missing data. As such, we could not exclude the possibility that survival benefit related to surgery and chemotherapy observed in this study may be inaccurate. Future studies using more accurate data are necessary to verify the results and better understand the survival impact of treatment.

Liver and lung are common sits of distant metastasis for CAC. Our analysis demonstrated that unmarried status, grade III/IV tumors, multiple regional lymph node metastasis, and elevated CEA were independent predictors for poor survival both in patients with liver‐only and lung‐only metastasis. The results highlighted CEA as an independent prognostic factor for stage IV disease, independent of TNM stage and for both liver‐only and lung‐only metastasis. This finding is consistent with previous reports,^35^, ^36^ that argue for routine preoperative CEA testing in patients with colon cancer to aid in treatment planning and prognosis assessment. Notably, our results revealed that the distal colon was associated with prolonged OS in patients with liver‐only metastasis, but not in patients with lung‐only metastasis, suggesting a disparity that may exist among patients with colon cancer. This is in accordance with previous studies indicating that cancers of the proximal and distal colon are distinct entities differing in embryologic origin, tumor behavior, genetic profile, and survival.^37^, ^38^ CEA is used clinically as a biomarker for CRC diagnosis and has been closely related to liver metastasis.^39^

This study has certain limitations. First, it should be noted that this analysis only included synchronous metastases diagnosed with the initial colon cancer. Consequently, this analysis might underestimate the metastatic burden of colon cancer and the results of metastatic patterns observed in this study might not be extended to patients with metachronous metastasis. Secondly, because the SEER database only recorded four sites of metastasis at diagnosis, this analysis did not comprehensively characterize site of metastasis from colon cancer. In addition, SEER provides limited information on treatment regimens, including details of adjuvant chemotherapy and surgery on metastasis. Third, the possible bias related to unable to accurately distinguish between no treatment and unknown if patients received treatment might lead to misleading results. However, multivariate analysis excluding treatment variables did not substantially change the results and led to the same conclusions of survival impact of metastatic patterns (results not shown). Fourth, the numbers of patients with brain and bone metastasis were relatively small, which reduced the statistical power to detect survival effects associated with these metastatic sites after adjustment for covariates. Future studies including more patients with brain and bone metastasis from colon cancer are needed to verify the results. In addition, subgroup analysis in patients with SRCC was not conducted due to limited sample size. Fifth, the vast majority of patients analyzed in this study were non‐Hispanic whites and thus, the results might not be generalizable to populations of different ethnic origins.

Despite these limitations, our study analyzed the largest cohort of patients with metastatic colon cancer and the results clearly indicated that site of distant metastasis was an independent prognostic factor for survival of patients with CAC. Surgical resection of the primary tumor and chemotherapy offered significant survival benefits to patients with metastatic disease. Staging system taking into account the site of metastasis may result in better treatment risk stratification and more accurate prediction of survival in patients with colon cancer.

## CONFLICT OF INTEREST

None declared.

## Data Availability

The data that support the findings of this study are available from the corresponding author upon reasonable request.
